# Lecithin nano-liposomal particle as a CRISPR/Cas9 complex delivery system for treating type 2 diabetes

**DOI:** 10.1186/s12951-019-0452-8

**Published:** 2019-01-29

**Authors:** Eun Yi Cho, Jee-Yeon Ryu, Han A. Reum Lee, Shin Hee Hong, Hye Sun Park, Kwan Soo Hong, Sang-Gyu Park, Hong Pyo Kim, Tae-Jong Yoon

**Affiliations:** 1 College of Pharmacy, Research Institute of Pharmaceutical Science and Technology (RIPST), Ajou University, 206 Worldcup-ro, Yeongtong-gu, Suwon, 16499 South Korea; 2 Bioimaging Research Team, Korea Basic Science Institute, Cheongju, 28119 South Korea; 3 Moogene Medi Co. Ltd., Korea Bio Park, Daewangpangyo-ro 700, Seongnam, 13488 South Korea

**Keywords:** CRISPR-Cas system, Nanoliposome, Type 2 diabetes mellitus, Dipeptidyl peptidase-4 gene

## Abstract

**Background:**

Protein-based Cas9 in vivo gene editing therapeutics have practical limitations owing to their instability and low efficacy. To overcome these obstacles and improve stability, we designed a nanocarrier primarily consisting of lecithin that can efficiently target liver disease and encapsulate complexes of Cas9 with a single-stranded guide RNA (sgRNA) ribonucleoprotein (Cas9-RNP) through polymer fusion self-assembly.

**Results:**

In this study, we optimized an sgRNA sequence specifically for dipeptidyl peptidase-4 gene (*DPP*-*4*) to modulate the function of glucagon-like peptide 1. We then injected our nanocarrier Cas9-RNP complexes directly into type 2 diabetes mellitus (T2DM) *db*/*db* mice, which disrupted the expression of *DPP*-*4* gene in T2DM mice with remarkable efficacy. The decline in DPP-4 enzyme activity was also accompanied by normalized blood glucose levels, insulin response, and reduced liver and kidney damage. These outcomes were found to be similar to those of sitagliptin, the current chemical DPP-4 inhibition therapy drug which requires recurrent doses.

**Conclusions:**

Our results demonstrate that a nano-liposomal carrier system with therapeutic Cas9-RNP has great potential as a platform to improve genomic editing therapies for human liver diseases.

**Electronic supplementary material:**

The online version of this article (10.1186/s12951-019-0452-8) contains supplementary material, which is available to authorized users.

## Background

Clustered regularly interspaced short palindromic repeats (CRISPR) and associated protein (Cas) nuclease complexes have been established as tools for human genome engineering therapy [1]. They are expected to facilitate drastic changes in the treatment of intractable genetic diseases [2]. However, conventional methods of transfecting cells with plasmid DNA to encode single-stranded guide RNA (sgRNA) and Cas nuclease exhibit critical limitations such as off-targeting, integration of foreign DNA, and toxicity due to the transfection agents [3, 4]. Direct delivery of protein-based nucleases such as Cas9 or Cpf1 with sgRNA complexes is another possible therapeutic strategy, but it faces low encapsulation efficiency, poor cell membrane permeability, and proteolytic instability, especially in vivo [5, 6]. As such, there is great need for a biocompatible vesicle carrier that can effectively encapsulate protein/sgRNA complexes. Liposomal particles are great candidate materials given their simple preparation method, easy surface modification, and high biocompatibility. For example, Wang et al. [7] have recently reported a bio-reducible lipid nanocarrier complex for protein-based Cas9 genome editing. It was produced through electrostatic interaction of cationic lipids and super-negatively charged complexes via protein–protein fusion.

In this study, type 2 diabetes mellitus (T2DM) was used as a target disease given its suitability for genetic therapy concerning liver. T2DM is a complex disease characterized by high glucose levels in the bloodstream, reduced glucose processing capacity in adipocytes, and insulin resistance in the body [8]. Elevating glucagon-like peptide-1 (GLP-1), an important target hormone that stimulates insulin secretion, is one of recent therapeutic approaches [9]. However, this hormone has a short half-life due to its extremely rapid degradation by enzyme dipeptidyl peptidase-4 (DPP-4) [10]. To prevent GLP-1 degradation, various drugs inhibiting DPP-4 such as sitagliptin, vildagliptin, saxagliptin, and linagliptin have been developed for insulin-mediated glucose control of T2DM [11]. Moreover, since DPP-4 inhibitors are widely used in clinical practice, they are also investigated as potential new therapeutics against the development of hepatic fibrosis and steatosis [12]. However, small-molecule antidiabetic drugs must be administered daily. In addition, they are associated with adverse effects such as hepatic impairment. A therapeutic method capable of effectively down-regulating DPP-4 enzyme with low side effects, such as a CRISPR/Cas9-based solution, would be an appropriate remedy for T2DM.

We prepared recombinant Cas9 nuclease complexes with an sgRNA as a ribonucleoprotein (Cas9-RNP) designed to edit the *DPP*-*4* gene. To deliver the Cas9-RNP complex, a lecithin-based liposomal nanocarrier particle (NL) was developed. To increase encapsulation efficiency, a cationic polymer was integrated with the Cas9-RNP complex to compensate for the NL’s negatively charged lipid structure. This is because loading efficiency is strongly dependent on electrostatic interactions [13]. Moreover, in consideration of biodistribution, NL are suitable for targeting liver diseases due to the natural metabolism of lecithin in the liver. Effects of Cas9-RNP incorporated NL were demonstrated by observing glucose tolerance and insulin resistance in T2DM mice.

## Methods

### Materials

Lecithin, cholesterol, rhodamine-B-Isothiocyanate (RITC), dimethyl sulfoxide (DMSO), and isopropyl β-D-1-thiogalactopyranoside (IPTG) were purchased from Sigma-Aldrich. DOGS-NTA-Ni was purchased from Avanti Polar Lipids. ICG-NHS was purchased from Goryo Chemicals. All chemicals were used directly without any purification.

### Synthesis of various NL particles

To synthesize NL particles, 1 mM lecithin, 0.7 mM cholesterol, and 0.3 mM DOGS-NTA-Ni were dissolved in 10 mL chloroform at 1:0.7:0.3 molar ratio and homogeneously mixed. The solution was evaporated to remove organic solvent and formed a thin lipid film. Recombinant Cas9-RNP complex prepared in 2 ml PBS (250 nM Cas9) was added to the lipid film and shaken gently for re-dispersion. The resulting multi-lamellar vesicle solution was then subjected to three cycles of freeze-thawing and probe sonication at 4 °C. To prepare fluorescent NL, primary amine reactive 19 μM rhodamine-B-Isothiocyanate (RITC) or 3.2 μM ICG-NHS in dimethyl sulfoxide (DMSO) solution was mixed with Cas9-RNP reaction buffer solution (0.1 M NaHCO_3_, pH 8.3) for 1 h in the dark. The mixing solution was loaded onto a spin-column (Thermo-Fisher), spun at 10,000*g* for 5 min, and washed 3 times with 100 μL PBS buffer solution. The labeled Cas9-RNP on the membrane was collected and dissolved in 200 μL PBS.

### Synthesis of recombinant Cas9 protein and sgRNA complexation

*Escherichia coli* BL21 competent cells (RBC Bioscience) were transformed with a pET28-(a) vector encoding SpCas9-NLS fused to a 6×His-tag with a C-terminal NLS followed by a His-tag (Addgene). Cells were incubated at 28 °C overnight. Isopropyl β-D-1-thiogalactopyranoside (IPTG) was then added at 0.5 mM to induce Cas9 protein expression. These cells were collected by centrifugation and lysed in lysis buffer [20 mM Tris–HCl (pH 8.0), 300 mM NaCl, 20 mM imidazole, 1× protease inhibitor cocktail, 1 mg/mL lysozyme] by sonication. Soluble lysate was obtained by centrifugation at 20,000*g* for 20 min at 4 °C. The soluble fraction was incubated with a column containing Ni–NTA agarose resin (Qiagen) at 4 °C to capture His-tagged Cas9 protein. Column-bound Cas9 protein was eluted with elution buffer [20 mM Tris–HCl (pH 8.0), 300 mM NaCl, 250 mM imidazole and 1 × protease inhibitor cocktail] and dialyzed in a storage buffer [50 mM Tris–HCl (pH 8.0), 200 mM KCl, 0.1 mM EDTA, 1 mM DTT, 0.5 mM PMSF, 20% glycerol]. Purification fractions of Cas9 protein were quantified using a bicinchoninic acid (BCA) assay kit (Pierce Biotechnology) and analyzed by SDS-PAGE and Coomassie staining. To prepare Cas9 and sgRNA complex with PEI, 75 μg Cas9 in 0.5 mL PBS buffer was homogenized with sgRNA solution (26 μg sgRNA with 0.5 mL PBS). Then 160 μg PEI (2 kDa) was incubated with the protein solution for 10 min.

### In vitro transcription of sgRNA

DNA fragment templates for sgRNA transcription were generated using an oligo containing T7 promoter and 20-bp sgRNA-specific target sequence overlapping with a second reverse complement oligo containing the constant region of the sgRNA backbone. All sgRNAs were transcribed using Guide-it™ sgRNA Screening kit (Clontech). In vitro transcribed RNA was extracted with phenol:chloroform:isoamyl alcohol (25:24:1, *v/v/v*), saturated with 10 nM Tris (pH 8.0) and 1 mM EDTA, precipitated with 2-propanol, and then re-dissolved in water. sgRNA concentration was quantified using a NanoDrop spectrophotometer and visualized after agarose gel electrophoresis.

### Intracellular delivery of NL particles

SNU398 cells were seeded into 60 mm^2^ dishes before the experiment. The prepared NL solution (250 nM Cas9) was mixed with FBS-free medium and added to cells. Cells were incubated at 37 °C for 2 h. After the 2 h incubation, excess nano-liposomes were removed, and treated cells were incubated in FBS-containing medium. To study the uptake mechanism of NL@Cas9-RNP, SNU398 cells were pretreated with 200 μM genistein, 30 μM chlorpromazine, 50 μM nocodazole, and 0.01% sodium azide or 5 μM cytochalasin B for 30 min at 37 °C or 4 °C followed by treatment with the NL solution (250 nM Cas9).

### In vitro cell analysis after treatment of NL@Cas9-RNP

For confocal laser scanning microscopy (CLSM) imaging of intracellular uptake of NL particles, SNU398 cells were seeded at density of 5000 cells/well into 4-well chamber slides (SPL) before the experiment. After treatment with RITC-labeled NL@Cas9-RNP for 2 h, treated cells were fixed with 4% paraformaldehyde and incubated in 1% BSA blocking buffer. Cells were then washed twice with PBS and incubated with anti-Cas9 (Abcam). After primary antibody incubation, cells were washed with PBS-T and incubated with CFL488-coupled secondary antibodies at room temperature for 1 h in the dark. At the end of incubation, cells were washed with TBS-T three times and counterstained with DAPI (Fluoroshield with DAPI mounting media). CLSM images were captured with a Leica TCS-SP5 laser scanning confocal microscope equipped with an oil objective.

### DNA sequencing and efficiency analysis

Genomic DNA was isolated from cells (SNU398) or mouse liver tissue using genomic isolation kit (Bioneer) according to the manufacturer’s instruction. PCR amplicons including nuclease target sites were generated using primers listed in Additional file 1: Figures S7a and S10b. To analyze DNA sequences, amplified PCR products corresponding to genomic modification were purified using PCR purification kit (Macherey–Nagel) and cloned into T-Blunt vector using PCR cloning kit (SolGent). Cloned products were sequenced by dye-terminator sequencing method (Bioneer). To analyze genomic editing efficiency, PCR products were purified with a PCR purification kit (Macherey–Nagel) and quantified. Purified PCR products were subsequently combined, denatured, and then annealed through thermos-cycling for 5 min (95 to 85 °C at 2 °C/s; 85 to 20 °C at 0.2 °C/s). Annealed DNA was incubated with T7 endonuclease I (T7EI, New England Biolabs) for 30 min at 37 °C and analyzed by 2% agarose gel electrophoresis.

### In vivo study for T2DM therapy

C57BL/6 wild-type mice and homozygous leptin receptor deficient *db*/*db* mice were purchased from Charles River Laboratories Japan, Inc. Mice were housed in a temperature- and humidity-controlled facility with a 12-h light/dark cycle for adaptation. To deliver particle under optimized conditions, 200 μL NL@Cas9-RNP (50 nM Cas9) was incubated at room temperature for 30 min before each injection. Sitagliptin (50 mM) was given daily to its treatment group by oral administration. Five mice were used for each treatment group. An EMD Millipore Multiplex MAP kit was used to assess concentrations of GLP-1 at the end of the 28-day treatment.

### In vivo monitoring of NL@Cas9-RNP

Optical imaging was performed post injection of ICG-labeled NL@Cas9-RNP using a homemade high-quality in vivo imaging system connected to an EMCCD camera (DU897-EX, iXon Ultra; Andor Technology). Fluorescence images of mice were acquired using an 808-nm (0–15 W) diode laser (OCLA) as an excitation light source and an emission filter (840 ± 12 nm, Semrock). Prior to imaging experiments, mice were anesthetized with 4% isoflurane/O_2_ (v/v) and maintained in 1–2% (v/v) isoflurane/O_2_ atmosphere throughout the experiment.

### In vivo therapeutic effect

Oral glucose tolerance test (OGTT) was performed at the end of the study. We carried out OGTT by taking baseline blood sample (200-μL) from the tail under local anesthesia and then gavaging with 50% glucose solution (Gibco) at 12 mL/kg to deliver 6 g of glucose per kg of body weight. Blood samples were taken at 30, 60, 120, and 180 min after the glucose meal and analyzed for blood glucose using Accu-Chek (Roche). For insulin tolerance test (ITT), regular human insulin (0.75 U/kg, Eli Lilly) was intraperitoneally injected and then blood samples were taken at 15, 30, 45, and 60 min post injection.

### Immunohistochemistry staining

At 28 days after treatment, mice were euthanized and then liver and pancreas were harvested. For immunochemistry staining, tissues were fixed with 4% paraformaldehyde at 4 °C overnight. Paraffin-embedded tissue sections were then cut into 5 μm thick sections and dried at 60 °C for 1 h. These sections were deparaffinized in xylene and rehydrated through graded ethanol solutions followed by rehydration with water. Antigen detection was performed using anti-DPP-4 (Origene) and anti-insulin (Abcam). After rinsing with tap water, sections were stained with hematoxylin and cover-slipped using a mounting medium.

## Results and discussion

### Screening sgRNA sequences for DPP-4 gene targeting

To ensure high gene targeting efficiency, we designed sgRNA sequences and delivered Cas9/sgRNA plasmids into SNU398 human liver carcinoma cells. We cloned *Streptococcus pyogenes* Cas9 (SpCas9) vectors co-expressing one of the sgRNA options that recognize the *DPP*-*4* gene and grafted a nuclear localization signal (NLS) sequence as well to enter the cell nucleus (Additional file 1: Figure S1a) [14]. Cells transfected with Cas9/sgRNA plasmids were differentiated based on mRNA expression, protein level, and enzyme activity using quantitative real-time PCR, western blotting, and DPP-4 enzyme activity assay, respectively (Additional file 1: Figure S1b–d). A further validation study showed that sgRNA2 targeting *DPP*-*4* gene worked better than sgRNA1 in decreasing mRNA, protein expression, and enzyme activity.

### Preparation of recombinant Cas9 protein nuclease and complexing with sgRNA

Recombinant Cas9 protein was prepared following a standard procedure previously reported [15]. Recombinant Cas9 protein containing NLS was purified from *Escherichia coli* and complexed with in vitro-transcribed sgRNA for targeting the *DPP*-*4* gene (Additional file 1: Table S1 and Figure S2a, b). We then monitored cleavage of the *DPP*-*4* gene caused by the prepared Cas9-RNP complex using an in vitro cleavage assay. Fragments (500 bp) of *DPP*-*4* genomic DNA were allowed to react with Cas9 protein alone or Cas9-RNP complex for 1 h. The cleavage was then determined based on agarose gel electrophoresis (Additional file 1: Figure S2c). Cas9 protein alone did not affect the genomic fragment. However, the Cas9-RNP complex showed clear gene editing activity, indicating target *DPP*-*4* gene recognition by the sgRNA complexed with Cas9 protein.

### Preparation of NL and encapsulation of the Cas9-RNP complex

**Fig. 1:**
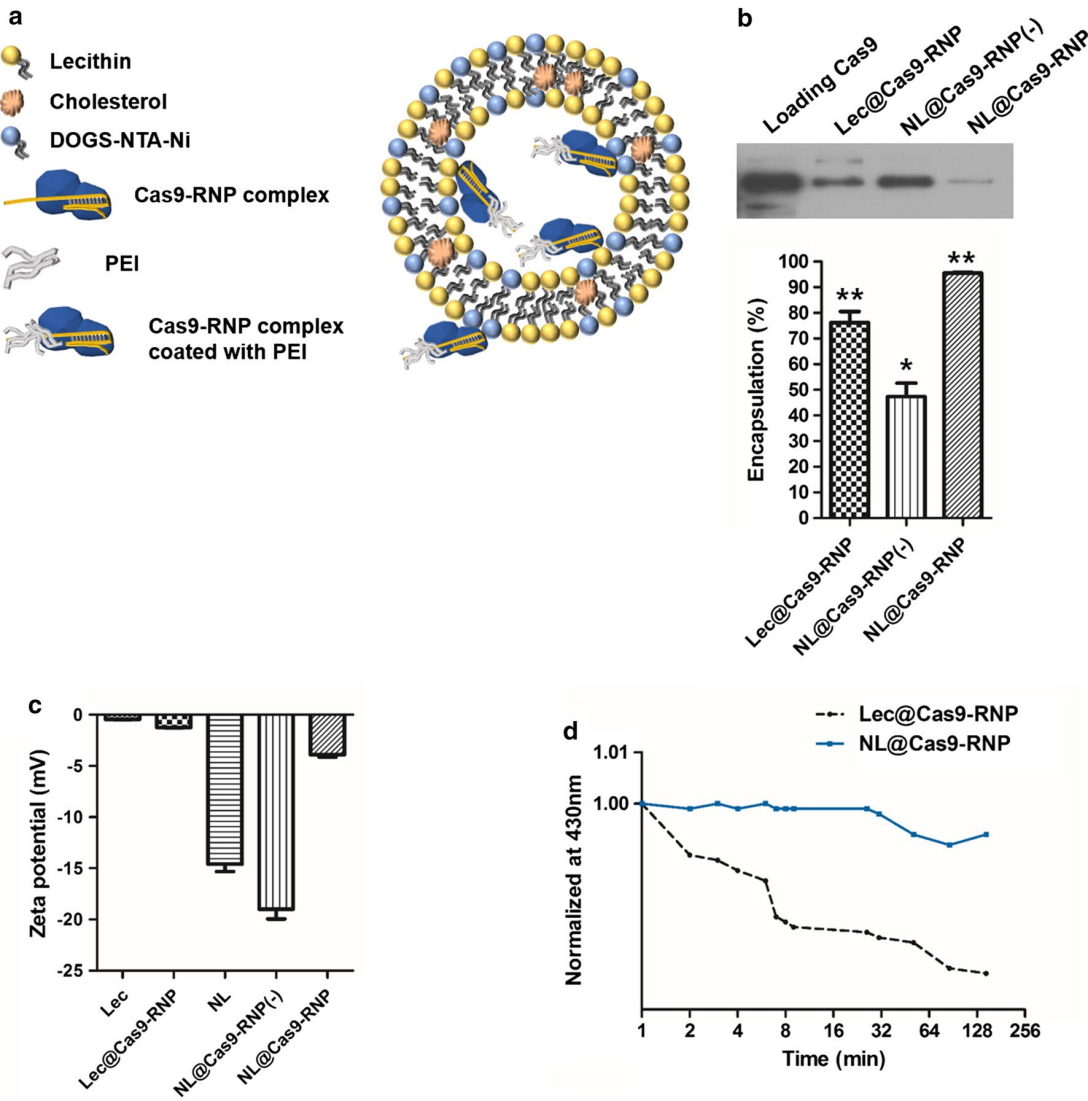
Characterization of various prepared liposomal particles. **a** Schematic of the prepared NL@Cas9-RNP particle system. **b** Measurement of encapsulation efficiency for various liposomal particles by assaying Cas9 protein level in the supernatant after centrifugation (upper panel shows western blotting data of supernatant, n = 3; *P < 0.05, **P < 0.01). **c** Zeta-potential analysis data of various particles. Lec: lecithin only liposome particle; Lec@Cas9-RNP: Cas9-RNP encapsulated Lec; NL@Cas9-RNP(−): Cas9-RNP encapsulated NL without PEI polymer fusion; NL@Cas9-RNP: PEI polymer linked Cas9-RNP encapsulated NL. **d** Determination of solution stability over time by measuring absorption at 430 nm through UV–Vis spectroscopy

The NL are mainly composed of biocompatible lecithin from egg yolk, which consists of phosphatidylcholine commonly found in human cell membranes [16]. As part of the NL, 1,2-dioleoyl-sn-glycerol-3-[(*N*-(5-amino-1-carboxylpentyl)iminodiacetic acid)succinyl]-(nickel salt) (shortened name: DOGS-NTA-Ni) was incorporated to mediate encapsulation by binding with His-tagged Cas9 proteins. NL particles of ~ 220 nm in size formed a spherical shape and a lipid bilayer encapsulating an aqueous soluble hydrophilic moiety by self-assembly. Lipid compounds were homogenously dissolved in a volatile hydrophobic organic solvent and then re-dispersed in a buffer solution containing the Cas9-RNP complex after evaporation of the organic solvent (Fig. 1a). The shape, size, and surface charge of formed NL were characterized using cryo-electron microscopy (Cryo-EM), dynamic light scattering (DLS), and zeta-potential analysis, respectively (Additional file 1: Figure S3). We optimized compositions of the NL particle carrier system after measuring the encapsulation efficiency of Cas9-RNP complexes. NL particles comprised of lecithin and DOGS-NTA-Ni without positive polymer fusion NL@Cas9-RNP(−), showed lower encapsulation efficiency of Cas9-RNP complexes compared to lecithin-alone particle (Lec) despite interaction between NTA-Ni and His-tag (Fig. 1b). Such difference in encapsulation efficiency can be due to repulsive electrostatic interactions between lipid compounds and Cas9-RNP complexes. Based on zeta-potential analysis, Lec particles without DOGS-NTA-Ni had less negative charge (− 0.438 mV) than NL particles (− 14.6 mV). From the zeta-potential analysis of Lec-alone particles and Lec encapsulated complex particles (Lec@Cas9-RNP), we also noticed that Cas9-RNP complexes had a slightly negative charge (Fig. 1c). Therefore, NL exhibited less incorporation efficiency than Lec-alone particle due to negative–negative repulsion. To increase the encapsulation efficiency, positively charged polyethyleneimine (PEI, 2 kDa) was utilized as a fusion material in the Cas9-RNP complex to compensate for its negative charge. PEI-fused Cas9-RNP complexes could be incorporated into the NL at a high capacity (NL@Cas9-RNP, 95%). They showed long-term solution stability without any aggregation (Fig. 1d). These NL particles appeared to maintain liposomal structure assembly well owing to interaction between NTA-Ni and His-tagged Cas9 protein as mentioned. However, Lec particles without DOGS-NTA-Ni exhibited destruction or sedimentation over time in solution (Additional file 1: Figure S3b, c). Taken together, these results showed that the use of PEI polymer fusion could increase the encapsulation efficiency and dispersion stability of protein-based Cas9-RNP complexes into NL.

### Gene editing in human cells by NL@Cas9-RNP

**Fig. 2:**
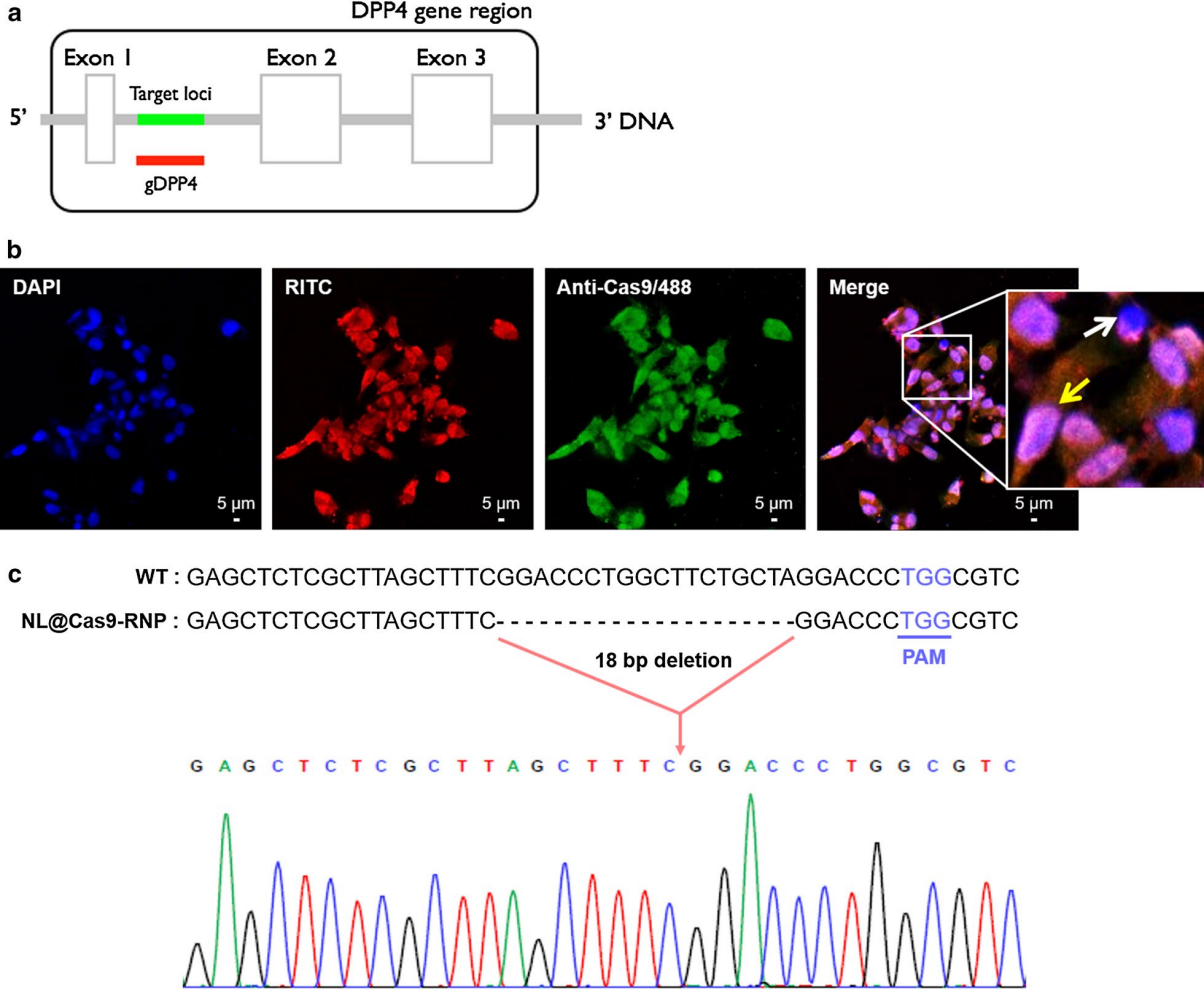
Delivery of NL@Cas9-RNP particles in a cell. **a** Schematic diagram describing the location of sgRNA to target *DPP*-*4* gene locus. **b** CLSM analysis of NL@Cas9-RNP cell uptake after labeling Cas9 with RITC to display protein distribution. Blue (DAPI) indicates the nucleus; Red (RITC-labeled Cas9-RNP) and green (anti-Cas9) are for Cas9 protein; Yellow arrow indicates co-localization of Cas9 and DAPI (i.e., overlapping of green and blue) which provides evidence for the penetration of Cas9 complex into the nucleus; White arrow indicates the absence of Cas9 protein. **c** Representation of sequence deletion at human *DPP*-*4* gene locus in SNU398 cells after gene editing by Bigdye-terminator sequencing

We first performed an in vitro cleavage assay to examine the activity of Cas9-RNP complex delivered by NL. Transcribed sgRNA from the sequence was designed to specifically recognize the promoter gene sequence between exon 1 and exon 2 of the *DPP*-*4* gene (Fig. 2a). To monitor cellular uptake, NL particles with fluorescent rhodamine B isothiocyanate (RITC)-labeled Cas9-RNP were incubated with SNU398 human liver carcinoma cells for 2 h. Subsequently, Cas9 protein staining agent to indicate the delivered Cas9 protein was added to cells and the localization of Cas9-RNP complex was analyzed by confocal laser scanning microscopy (CLSM) (Fig. 2b). NL@Cas9-RNP particles were dispersed in the cytosol. Green-stained Cas9 proteins were co-localized with these particles in the cytosol as well as in the nucleus which was stained with DAPI. To understand the uptake mechanism of NL@Cas9-RNP, SNU398 cells were stained with anti-Cas9-488 after various inhibitors at different temperatures for 24 h (Additional file 1: Figure S4). Particle internalization into cells was negatively affected by pretreatment with genistein (clathrin endocytosis inhibitor), sodium azide (metabolic/ATP inhibitor), cytochalasin B (phagocytosis inhibitor), nocodazole (macropinocytosis inhibitor), and low temperature incubation (4 °C). Conversely, particles were normally internalized after pre-treatment of cells with chlorpromazine (caveolin-mediated endocytosis inhibitor). These results indicate that the cellular uptake mechanism of NL@Cas9-RNP particles mainly involves clathrin-mediated energy dependent phagocytosis and macropinocytosis. We then evaluated in vitro *DPP*-*4* gene editing ability of NL@Cas9-RNP in human cells. Cas9-mediated gene deletion junctions (18 bp) at the *DPP*-*4* locus were confirmed by sequencing analysis (Fig. 2c). Based on sequencing results, it was apparent that *DPP*-*4* gene disruption junctions were created upstream of the protospacer adjacent motif (PAM) sequence. NL@Cas9-RNP exhibited the highest inhibition for DPP-4 enzyme production through gene editing. The inhibition efficiency was compared to other particles using an equal concentration of Cas9-RNP complex (Additional file 1: Figure S5). We surmise that the enhanced editing capability of NL@Cas9-RNP is likely due to increased solution stability and reduced negative surface charge. Next, we verified that *DPP*-*4* gene disruption did not cause cellular toxicity or stress using a cell viability and a cell stress assay kit, respectively (Additional file 1: Figure S6). The various carrier particles we synthesized did not show cytotoxicity under culture conditions. The delivery system did not affect cell proliferation or differentiation. Our results confirmed that most cellular stress bio-markers expressed no significantly different between treated and untreated cells. A slight increase in hypoxia-inducible factor 2 (HIF-2), an oxidative stress response component, and paraoxonase 1 (Pon1), a lipid metabolism component, were observed. However, these results have negligible impact on overall cell viability. These results confirmed that NL-based Cas9-RNP carrier system has low cytotoxicity and high biocompatibility in a cell.

### Off-target cleavage effects of NL@Cas9-RNP in cells

To evaluate gene editing specificity, we assessed the capability of NL@Cas9-RNP to reduce genome-wide off-target effects at different positions depending on various target sequences of chromosomes (Additional file 1: Figure S7a). In vitro genomic editing efficiency was found to be ~ 31% by T7 endonuclease I (T7EI) assay. We exploited seven different genomic off-target sites with 3- to 5-bases mismatched sequences of chromosome (Additional file 1: Figure S7b). Results showed that NL@Cas9-RNP was highly specific to *DPP*-*4* gene sequence as an on-target site. These results demonstrated that our protein-based gene editing system could effectively recognize target sequences with high specificity, thus substantially improving sgRNAs targeting to homopolymeric sequences.

### In vivo gene editing by NL@Cas9/gDPP-4 delivery in a T2DM mouse model

**Fig. 3:**
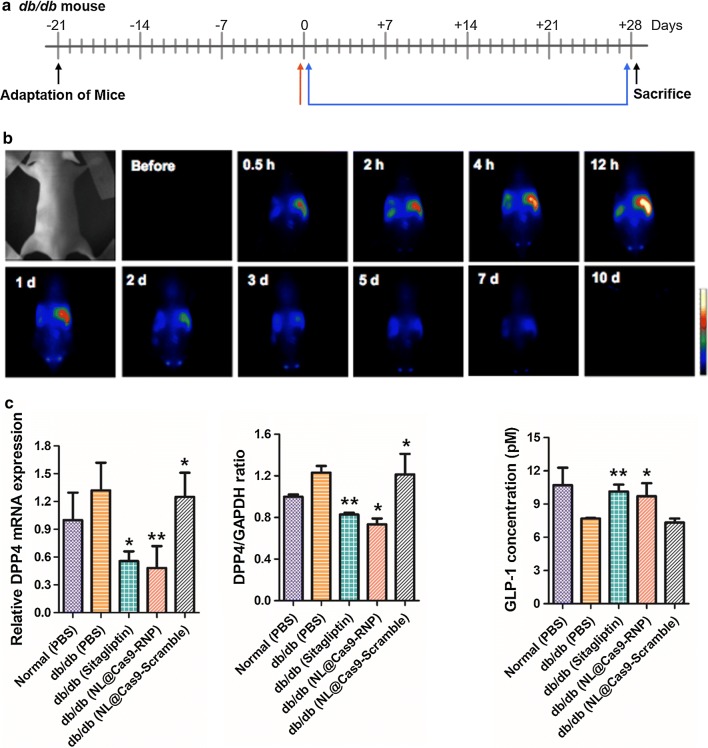
*In vivo* therapeutic effect. **a** Schedule diagram showing therapeutic effect assessment. All mice were adapted for 21 days. Sitagliptin was given daily via oral administration for 28 days (blue arrow region) while NL@Cas9-RNP particles were injected intravenously by a single administration (red arrow). **b** NL@Cas9-RNP with NIR dye was monitored to assess bio-distribution at different times by optical imaging. Only dye-conjugated Cas9 protein injection data are presented in Additional file 1: Figure S8. **c** Expression levels of *DPP*-*4* mRNA (left) and protein (right) in extracted liver tissue were determined by quantitative real-time PCR and western blotting, respectively. **d** Serum concentration of GLP-1 from treated mice was examined quantitatively using an assay kit. (n = 3, *P < 0.05 and **P < 0.01)

**Fig. 4:**
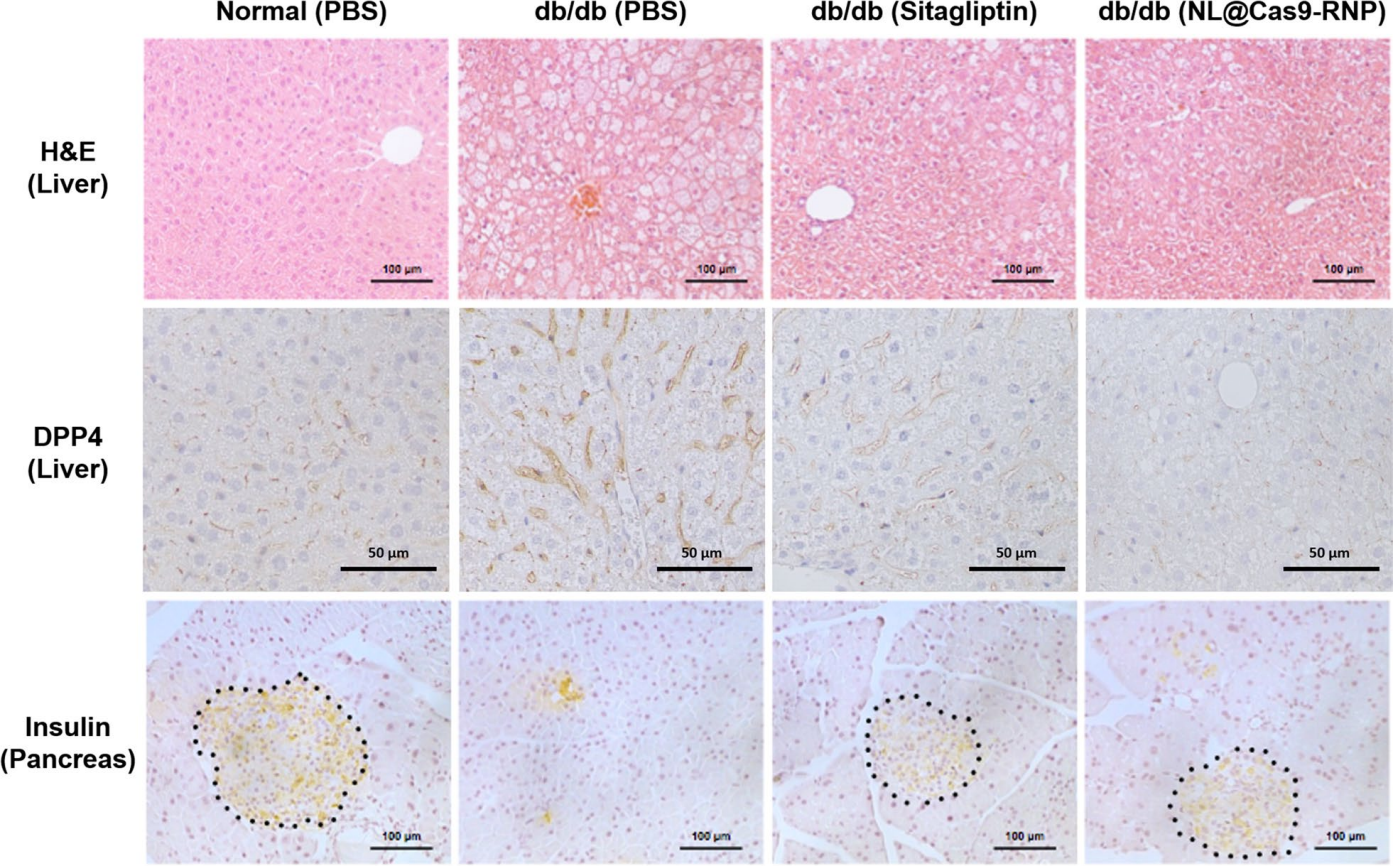
Histological analysis data. H&E and DPP-4 staining of liver tissue and insulin staining images of pancreas tissue after 28-days treatment. Dotted circles indicate islet cores

We demonstrated the in vivo delivery viability of NL@Cas9-RNP particle with an sgRNA tailored to target the mouse *DPP*-*4* gene sequence (m*DPP*-*4*) (Additional file 1: Table S1). We compared the therapeutic effect of NL@Cas9-RNP with sitagliptin, a clinically used drug. NL@Cas9-RNP in PBS was administered to T2DM *db*/*db* mice via intravenous injection after adaptation while sitagliptin was orally administered daily to *db*/*db* mice for 28 days (Fig. 3a). To monitor the in vivo biodistribution of NL@Cas9-RNP, Cas9-RNP was labeled with indocyanine green (ICG) near-infrared (NIR) dye before incorporation into the NL and the distribution of particles was determined over time by NIR imaging (Fig. 3b). As expected, the NL were observed to accumulate in the liver from 2 h to 1 day after injection. Accumulation of nanomaterials in the liver is likely attributable to macrophage recognition [17]. Conversely, dye-labeled bare Cas9 proteins showed different haphazard distribution pattern after injection into mouse. They were quickly degraded from the liver after injection (Additional file 1: Figure S8). After 12 h of administration, 81.3% of particles with dye labeled Cas9 complexes were delivered to the liver (Additional file 1: Figure S9). After liver harvest, genomic DNA fragment deletion assay of liver tissue showed clear gene editing efficiency of 39% and an off-target effect similar to that seen in vitro (Additional file 1: Figure S10). DPP-4 expression levels in liver tissues were also assessed using either qPCR or western blotting to evaluate the gene editing efficacy of NL@Cas9-RNP (Fig. 3c). In *db*/*db* PBS-injected mice and scrambled sgRNA of unrelated sequence containing particle injected control mice, we observed high *DPP*-*4* mRNA and enzyme expression in the liver. However, NL@Cas9-RNP administered *db/db* mice exhibited decreases for mRNA and protein levels of DPP-4. We also assessed *DPP*-*4* gene editing capacity by quantitatively examining serum concentrations of GLP-1 using an assay kit (Fig. 3d) [18]. Serum GLP-1 was decreased by 28% in PBS-injected *db*/*db* mice compared to that in control mice. Both sitagliptin and NL@Cas9-RNP treated mice showed dramatically increased serum GLP-1 concentrations (by 24% and 21%, respectively) compared to those in PBS-injected control *db*/*db* mice. When data iscompared across organ tissues, *DPP*-*4* mRNA expression levels were mainly decreased in the liver (Additional file 1: Figure S11). These results suggest that NL particles containing Cas9-RNP does not affect other organs during the metabolic process. H&E staining of liver tissue (Fig. 4) showed that PBS-injected control *db*/*db* mice had a high content of fat pores whereas NL@Cas9-RNP treated mice exhibited decreased fat deposition in the liver. Therefore, partial disruption of DPP-4 gene can also influence liver fat accumulation since the DPP-4 enzyme is involved in the regulation of hepatic metabolism and non-alcoholic fatty liver disease (NAFLD) in subjects with diabetes or obesity [19]. Histological analysis of the pancreas revealed that the islets of normal mice had high levels of insulin immune-reactivity while PBS-injected *db*/*db* control mice had altered islet morphology. The distribution of insulin-producing cells (*β*-cells) from the islet core of *db*/*db* mice was rarely detected. In contrast, sitagliptin or NL@Cas9-RNP treatment recovered normal islet clusters and morphology, suggesting normal insulin production. In addition, NL@Cas9-RNP treated mice showed decreased body weight, similar to sitagliptin-treated mice (Additional file 1: Figure S12).

### Blood analysis for glucose regulation and organ function test

**Fig. 5:**
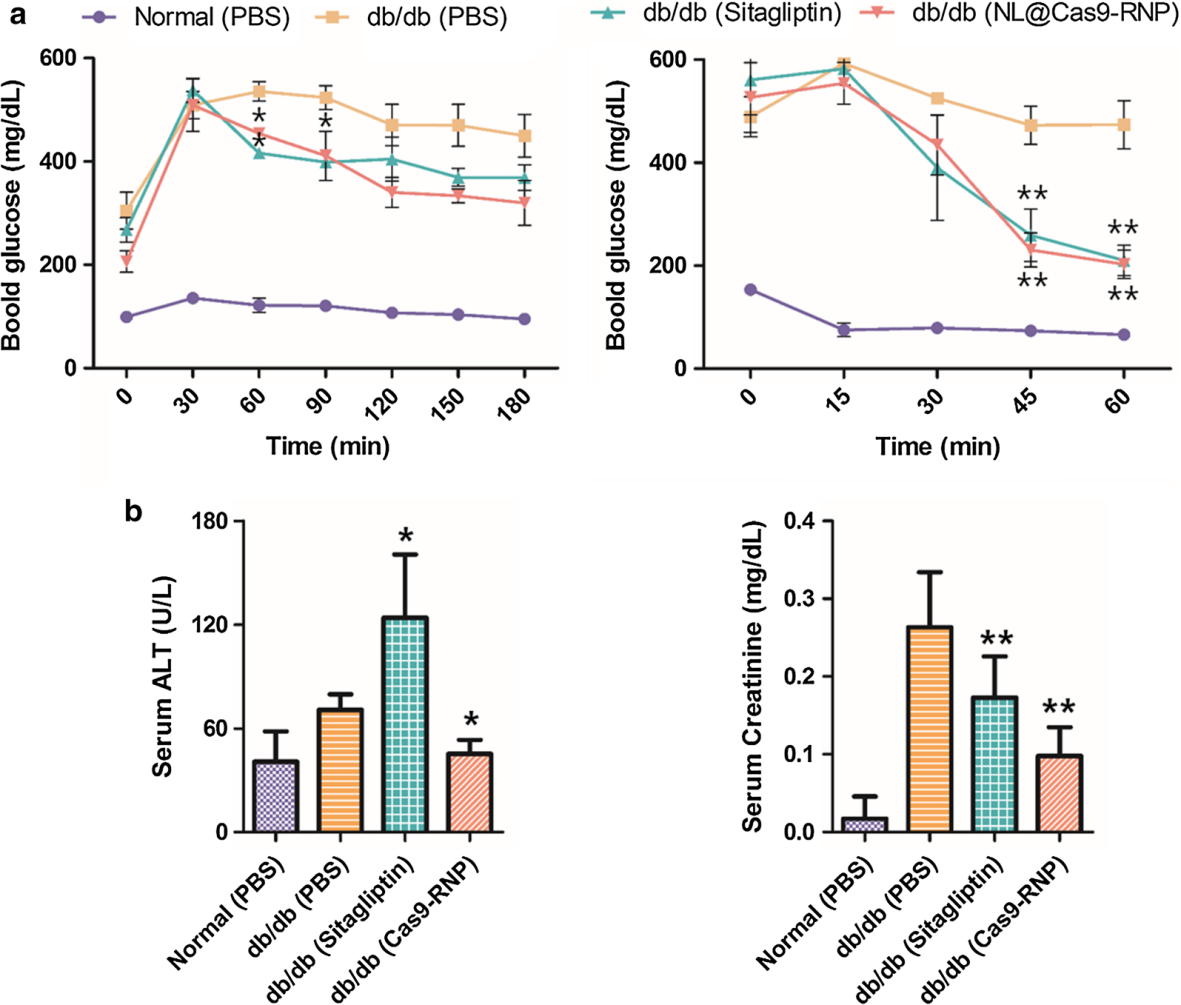
OGTT, ITT, and serological testing results. **a** OGTT and ITT values at various times after administration of glucose meal or insulin. **b** Measurement of serum ALT and renal creatinine levels to investigate liver and kidney function. n = 3; *P < 0.05, **P < 0.01, ***P < 0.001

In animals, oral glucose tolerance test (OGTT) can be used to indicate relative roles of insulin secretion and insulin resistance in the progression of glucose intolerance [20]. OGTT was performed in starved mice for 24 h. Glucose solution was administered orally and blood was serially sampled and analyzed for glucose concentration over time (Fig. 5a). The initial glucose concentration was as low as the fasting level. However, it was increased to a maximum value of approximately 520 mg/dL in *db*/*db* mice within 30 min. Blood glucose level in PBS-injected *db*/*db* mice remained above 450 mg/dL due to glucose tolerance. However, blood glucose levels in both NL@Cas9-RNP treated and sitagliptin-treated mice decreased gradually. After starving mice for 6 h, an intraperitoneal insulin tolerance test (ITT, injection of 0.75 IU/kg body weight of soluble insulin) showed no significant changes in glucose levels within 1 h for PBS-injected *db*/*db* mice (control). However, blood glucose levels in sitagliptin and NL@Cas9-RNP treated *db*/*db* mice were quickly decreased due to decline in insulin resistance. Although NL@Cas9-RNP was administered only once, it was sufficient for producing a therapeutic effect similar to daily sitagliptin-treatment for 28 days based on OGTT and ITT values. A spectrophotometric serological investigation was also conducted to measure serum levels of alanine aminotransferase (ALT) and creatinine, with high levels indicating functional impairment of the liver and kidney (Fig. 5b). Sitagliptin-treated mice showed higher levels of serum ALT and creatinine than PBS or NL@Cas9-RNP treated *db*/*db* mice. Daily administration of sitagliptin appeared to either increase hepatotoxicity and/or induce kidney dysfunction. Conversely, serum levels of these serological parameters in NL@Cas9-RNP treated mice were normal or even decreased, indicating negligible impact of NL@Cas9-RNP on liver or kidney function. In conclusion, NL@Cas9-RNP is demonstrated to be an effective therapeutic strategy for T2DM through *DPP*-*4* gene editing.

## Conclusion

Protein delivery for genome editing has undergone much recent development for therapeutic applications given its advantages such as low off-target effects and non-integration over plasmid delivery. Delivery of Cas9 in protein form has been previously attempted by other groups using methods such as local delivery, lipid nanoparticles, DNA nanoclew, and gold nanoparticle [21–24]. We implemented a nanocarrier system, mainly composed of lecithin, that could effectively deliver Cas9-RNP complexes into hepatocytes via accumulation in the liver. Here were report the synthesis and application of NL containing therapeutic Cas9-RNP complexes for genome editing in vitro and in vivo. The encapsulation of Cas9-RNP complexes was enhanced by increasing the loading efficiency through fusion of a positively charged polymer. Spontaneous electrostatic interaction between charge-compensated complexes and negatively charged lipids resulted in self-assembly of NL spheres with uniform size distribution. Unlike unprotected protein therapeutic strategies that have low delivery efficacy due to enzymatic breakdown, our genome platform has exemplary biocompatibility with low cytotoxicity and high solution stability, making it optimal for genetic and chronic human disease therapies. As proof of concept, T2DM was selected as disease of focus. We designed a therapeutic strategy whereby the *DPP*-*4* gene was disrupted by NL@Cas9-RNP delivery. We optimized an sgRNA sequence to target the *DPP*-*4* gene. NL@Cas9-RNP successfully edited this gene with very low cytotoxicity. In *db*/*db* mice, NL@Cas9-RNP particle was able to decrease levels of DPP-4. Such disruption induced up-regulation of GLP-1 concentration in the blood. The enhanced efficacy of the gene disruption effect might be attributed to extended retention of NL in the liver given that hepatocytes can effectively uptake lecithin-based NL particles. Down-regulation of DPP-4 by NL@Cas9-RNP treatment also reconciled glucose tolerance and insulin resistance in a manner similar to sitagliptin. Interestingly, blood analysis showed that unlike sitagliptin, NL@Cas9-RNP treatment maintained normal liver and kidney function probably because of lessened drug dispensation. NL@Cas9-RNP can be applied as an effective treatment option for mitigating T2DM and its development. Moreover, our therapeutic strategy using NL@Cas9-RNP to target *DPP*-*4* can be used for other diseases as well given that DPP-4 is known to exert substantial pleiotropic effects for a variety of diseases such as obesity, chronic renal disease, cardiovascular disease, and inflammation. Our results suggest that NL@Cas9-RNP system possesses several advantages: (1) highly efficient encapsulation of the Cas9-RNP complex, (2) effective and stable delivery in vivo, and (3) effective therapy concerning liver disease. More studies are warranted to characterize and optimize the pharmacokinetics, efficacy, and safety of such *DPP*-*4* gene editing strategy using NL@Cas9-RNP in animal models.

## Additional file

**Additional file 1.** Information of the sgRNA sequence for DPP-4 gene targeting, characterization data for the Cas9-RNP preparation, uptake mechanism and gene editing efficiency in vitro and in vivo results after treatment of NL(Cas9-RNP) particles. **Figure S1.** Design of sgRNA for human DPP4 gene targeting. (**a**) Schematic for a Cas9/sgRNA plasmid. We designed two different sequences of sgRNAs for human *DPP*-*4* gene. (**b**) The expression level of *DPP*-*4* mRNA was examined using quantitative real-time PCR. Cas9-RNP (sgRNA2) showed lower expression by ~ 69% relative to that of untreated control cells. (**c**) Corresponding DPP-4 enzyme level was reduced by 46% based on western blotting after treatment with Cas9-RNP (sgRNA2). (**d**) DPP-4 enzyme activity assessed with an assay kit. n = 3; *P < 0.05, **P < 0.01, ***P < 0.001. **Table S1.** List of primer sequences for in vitro sgRNA transcription. Designed sgRNA sequence for targeting DPP-4 gene region, we prepared sgRNAs from various primer sequences in the list by in vitro transcription. **Figure S2.** Purification and cleavage ability of the prepared Cas9-RNP complex. (**a**) 10% SDS-PAGE of recombinant Cas9 protein purified from *E. coli.* (**b**) Schematic representation of in vitro-transcribed sgRNAs composed of a 20-nucleotide guide sequence for *DPP*-*4* gene recognition and a scaffold sequence for complexing with Cas9 recombinant protein. (**c**) in vitro cleavage assay. Cas9-RNP showed clear cleavage of 500-bp target *DPP*-*4* gene. **Figure S3.** Characterization of the nano-liposomal particle. (**a**) Cryo-EM images of lecithin-alone particle (left, Lec) and NL particle (right) containing Cas9-RNP complexes. (**b)** DLS data of Lec@Cas9-RNP (scale bar = 200 nm). (**c**) DLS data of NL@Cas9-RNP. The diameter of Lec@Cas9-RNP ranged from 164.2 to 1718 nm while NL@Cas9-RNP showed a uniform size distribution with an average diameter of 220.2 nm. **Figure S4.** Uptake mechanism study of NL@Cas9-RNP particle into cells. Assessment of the uptake mechanism using green fluorescence staining of delivered Cas9 protein with anti-Cas9-488 antibodies under various conditions such as inhibitor treatment or culture temperature change. The nucleus is stained with DAPI (blue). SNU398 cells were pretreated for 30 min with various inhibitors: genistein (200 μM), chlorpromazine (30 μM), nocodazole (50 μM), sodium azide (0.01%), or cytochalasin B (5 μM) at 37 °C or 4 °C before NL@Cas9-RNP treatment (Scale bar = 50 μm). **Figure S5.** Gene editing efficiency in human cells. We investigated whether various nano-liposomes were able to perform gene editing by delivering Cas9-RNP into mammalian cells. SNU398 cells were treated with Lec@Cas9-RNP, NL@Cas9-RNP(-), or NL@Cas9-RNP. Expression levels of *DPP*-*4* mRNA and protein were measured using quantitative real-time PCR (**a**) and western blotting (**b**), respectively. NL@Cas9-RNP has the highest editing efficiency with decreased mRNA (67%) and enzyme protein (87%) expression. In particular, DPP-4 enzyme activity owing to NL@Cas9-RNP delivery was decreased by 48% compared to that of control SNU398 cells (**c**). n = 3; *P < 0.05, **P < 0.01, ***P < 0.001. **Figure S6.** Cell toxicity assessment of various particles. (**a**) Cell viability after treatment with various particles containing Cas9-RNP complexes using WST-1 assay. (**b**) Cell stress assessment after treatment using Human Cell Stress Array kit (R&D Systems). **Figure S7.** Off-target effects of NL@Cas9-RNP. (**a**) List of on- or off-target sequences with mismatch sites and mismatched bases shown in red. (**b**) On-target or potential off-target effects in various target sequence of chromosomes (Chr#) were identified by T7EI assay. Cas9-RNP shows deletion of the DNA on-target site in a cell. **Figure S8.** In vivo monitoring. NIR dye intensity of bare conjugated Cas9 proteins was monitored by optical imaging after injection. **Figure S9.** In vivo bio-distribution data. NL particles with dye-labeled Cas9-RNP complexes were used to determine bio-distribution in various organs. **Figure S10.** In vivo gene editing efficiency. (**a**) Representative sequence analysis of deletions at *DPP*-*4* locus in *db/db* mice after gene editing by Bigdye-terminator sequencing (see Methods). (**b**) List of various sequences of target sequences with mismatch sites and mismatched bases shown in red. (**c**) On-target and potential off-target effects in various target sequence of chromosomes were identified by T7EI assay. NL@Cas9-RNP achieved in vivo gene editing efficiency of 39% for DNA on-target site with low off-target effect in mouse liver. **Figure S11.** Comparison of *DPP*-*4* mRNA expression distribution in various organ tissues of *db/db* mice after treatment based on quantitative real-time PCR. **Figure S12.** Sitagliptin and NL@Cas9-RNP treated mice had relatively decreased body weight compared to control mice.

## Abbreviations

CRISPR: clustered regularly interspaced short palindromic repeats
sgRNA: single-stranded guide RNA
T2DM: type 2 diabetes mellitus
GLP-1: glucagon-like peptide-1
DPP-4: dipeptidyl peptidase-4 gene
NL: lecithin-based liposomal carrier particle
NLS: nuclear localization signal
DOGS-NTA-Ni: 1,2-dioleoyl-sn-glycerol-3-[(*N*-(5-amino-1-carboxylpentyl)iminodiacetic acid)succinyl]-(nickel salt)
Cryo-EM: cryo-electron microscopy
DLS: dynamic light scattering
Lec: lecithin-alone particle
PEI: polyethyleneimine
NL@Cas9-RNP: PEI-fused Cas9-RNP complexes into the NL
RITC: rhodamine B isothiocyanate
CLSM: confocal laser scanning microscopy
PAM: protospacer adjacent motif
HIF-2: hypoxia-inducible factor 2
Pon-1: paraoxonase 1
OGTT: oral glucose tolerance test
ITT: intraperitoneal insulin tolerance test
ALT: alanine aminotransferase

## References

[1] Cho SW, Kim S, Kim JM, Kim JS (2013). Targeted genome engineering in human cells with the Cas9 RNA-guided endonuclease. Nat Biotechnol.

[2] Ghorbal M, Gorman M, Macpherson CR, Martins RM, Scherf A, Lopez-Rubio JJ (2014). Genome editing in the human malaria parasite plasmodium falciparum using the CRISPR-Cas9 system. Nat Biotechnol.

[3] Fu Y, Foden JA, Khayter C, Maeder ML, Reyon D, Joung JK, Sander JD (2013). High-frequency off-target mutagenesis induced by CRISPR-Cas nucleases in human cells. Nat Biotechnol.

[4] Kotterman MA, Schaffer DV (2014). Engineering adeno-associated viruses for clinical gene therapy. Nat Rev Genet.

[5] Kim H, Kim ST, Ryu J, Kang BC, Kim JS, Kim SG (2017). CRISPR/Cpf1-mediated DNA-free plant genome editing. Nat Commun..

[6] Gu Z, Biswas A, Zhao M, Tang Y (2011). Tailoring nanocarriers for intracellular protein delivery. Chem Soc Rev.

[7] Wang M, Zuris JA, Meng F, Rees H, Sun S, Deng P, Han Y, Gao X, Pouli D, Wu Q, Georgakoudi I, Liu DR, Xu Q (2016). Efficient delivery of genome-editing proteins using bioreducible lipid nanoparticles. Proc Natl Acad Sci USA.

[8] Skyler JS (2004). Diabetes mellitus: pathogenesis and treatment strategies. J Med Chem.

[9] Baggio LL, Drucker DJ (2007). Biology of incretins: GLP-1 and GIP. Gastroenterology.

[10] Drucker DJ, Nauck MA (2006). The incretin system: glucagon-like peptide-1 receptor agonists and dipeptidyl peptidase-4 inhibitors in type 2 diabetes. Lancet.

[11] Rohrborn D, Wronkowitz N, Eckel J (2015). DPP-4 in diabetes. Front Immunol..

[12] Scheen AJ (2014). Pharmacokinetic and toxicological considerations for the treatment of diabetes in patients with liver disease. Expert Opin Drug Metab Toxicol..

[13] Pohl C, Kiel JA, Driessen AJ, Bovenberg RA, Nygard Y (2016). CRISPR/Cas9 based genome editing of penicillium chrysogenum. ACS Synth Biol..

[14] Kim S, Kim D, Cho SW, Kim J, Kim JS (2014). Highly efficient RNA-guided genome editing in human cells via delivery of purified Cas9 ribonucleoproteins. Genome Res.

[15] van Hoogevest P, Wendel A (2014). The use of natural and synthetic phospholipids as pharmaceutical excipients. Eur J Lipid Sci Technol.

[16] Liu J, Jiang X, Ashley C, Brinker CJ (2009). Electrostatically mediated liposome fusion and lipid exchange with a nanoparticle-supported bilayer for control of surface charge, drug containment and delivery. J Am Chem Soc.

[17] Kim M, Sonya AM, Xue-Zhong M, Vinzent NS, Jaun E, Ben O, Saleh MF, Edward AS, Nicolas G, Johann MK, John BC, Benjamin AA, Markus S, Mario AO, Oyedele AA, Anton Z, Ian DM, Warren C (2016). Mechanism of hard nanomaterial clearance by the liver. Nat Mater.

[18] Adrian TE, Gariballa S, Parekh KA, Thomas SA, Saadi H, Al Kaabi J, Nagelkerke N, Gedulin B, Young AA (2012). Rectal taurocholate increases L cell and insulin secretion and decreases blood glucose and food intake in obese type 2 diabetic volunteers. Diabetologia.

[19] Williams KH, De Vieira Ribeiro AJ, Prakoso E, Veillard AS, Shackel NA, Brooks B, Bu Y, Cavanagh E, Raleigh J, McLennan SV, McCaughan GW, Keane FM, Zekry A, Gorrell MD, Twigg SM (2015). Circulating dipeptidyl peptidase-4 activity correlates with measures of hepatocyte apoptosis and fibrosis in non-alcoholic fatty liver disease in type 2 diabetes mellitus and obesity: a dual cohort cross-sectional study. J Diabetes.

[20] Slingerland LI, Robben JH, van Haeften TW, Kooistra HS, Rijnberk A (2007). Insulin sensitivity and beta-cell function in healthy cats: assessment with the use of the hyperglycemic glucose clamp. Horm Metab Res.

[21] Staahl BT, Benekareddy M, Coulon-Bainier C, Banfal AA, Floor SN, Sabo JK, Urnes C, Munares GA, Ghosh A, Doudna JA (2017). Efficient genome editing in the mouse brain by local delivery of engineered Cas9 ribonucleoprotein complexes. Nat Biotechnol.

[22] Zuris JA, Thompson DB, Shu Y, Guilinger JP, Bessen JL, Hu JH, Maeder ML, Joung JK, Chen ZY, Liu DR (2015). Cationic lipid-mediated delivery of proteins enables efficient protein-based genome editing in vitro and in vivo. Nat Biotechnol.

[23] Mout R, Ray M, Yesilbag Tonga G, Lee YW, Tay T, Sasaki K, Rotello VM (2017). Direct cytosolic delivery of CRISPR/Cas9-ribonucleoprotein for efficient gene editing. ACS Nano.

[24] Sun W, Ji W, Hall JM, Hu Q, Wang C, Beisel CL, Gu Z (2015). Self-assembled DNA nanoclews for the efficient delivery of CRISPR/Cas9 for genome editing. Angew Chem Int Ed.

