# Supplementary material for: Lecithin nano-liposomal particle as a CRISPR/Cas9 complex delivery system for treating type 2 diabetes
Source: J Nanobiotechnology. 2019 Jan 29;17:19. doi: 10.1186/s12951-019-0452-8 (PMC6350399; doi:10.1186/s12951-019-0452-8)
Supplement: Supplementary file 1 — Additional file 1. Information of the sgRNA sequence for DPP-4 gene targeting, characterization data for the Cas9-RNP preparation, uptake mechanism and gene editing efficiency in vitro and in vivo results after treatment of NL(Cas9-RNP) particles. Figure S1. Design of sgRNA for human DPP4 gene targeting. (a) Schematic for a Cas9/sgRNA plasmid. We designed two different sequences of sgRNAs for human DPP-4 gene. (b) The expression level of DPP-4 mRNA was examined using quantitative real-time PCR. Cas9-RNP (sgRNA2) showed lower expression by ~ 69% relative to that of untreated control cells. (c) Corresponding DPP-4 enzyme level was reduced by 46% based on western blotting after treatment with Cas9-RNP (sgRNA2). (d) DPP-4 enzyme activity assessed with an assay kit. n = 3; *P < 0.05, **P < 0.01, ***P < 0.001. Table S1. List of primer sequences for in vitro sgRNA transcription. Designed sgRNA sequence for targeting DPP-4 gene region, we prepared sgRNAs from various primer sequences in the list by in vitro transcription. Figure S2. Purification and cleavage ability of the prepared Cas9-RNP complex. (a) 10% SDS-PAGE of recombinant Cas9 protein purified from E. coli. (b) Schematic representation of in vitro-transcribed sgRNAs composed of a 20-nucleotide guide sequence for DPP-4 gene recognition and a scaffold sequence for complexing with Cas9 recombinant protein. (c) in vitro cleavage assay. Cas9-RNP showed clear cleavage of 500-bp target DPP-4 gene. Figure S3. Characterization of the nano-liposomal particle. (a) Cryo-EM images of lecithin-alone particle (left, Lec) and NL particle (right) containing Cas9-RNP complexes. (b) DLS data of Lec@Cas9-RNP (scale bar = 200 nm). (c) DLS data of NL@Cas9-RNP. The diameter of Lec@Cas9-RNP ranged from 164.2 to 1718 nm while NL@Cas9-RNP showed a uniform size distribution with an average diameter of 220.2 nm. Figure S4. Uptake mechanism study of NL@Cas9-RNP particle into cells. Assessment of the uptake mechanism using green fluorescence st [file 12951_2019_452_MOESM1_ESM.docx]

**Additional File 1**

**Lecithin nano-liposomal particle as a CRISPR/Cas9 complex delivery system for treating type 2 diabetes**

**
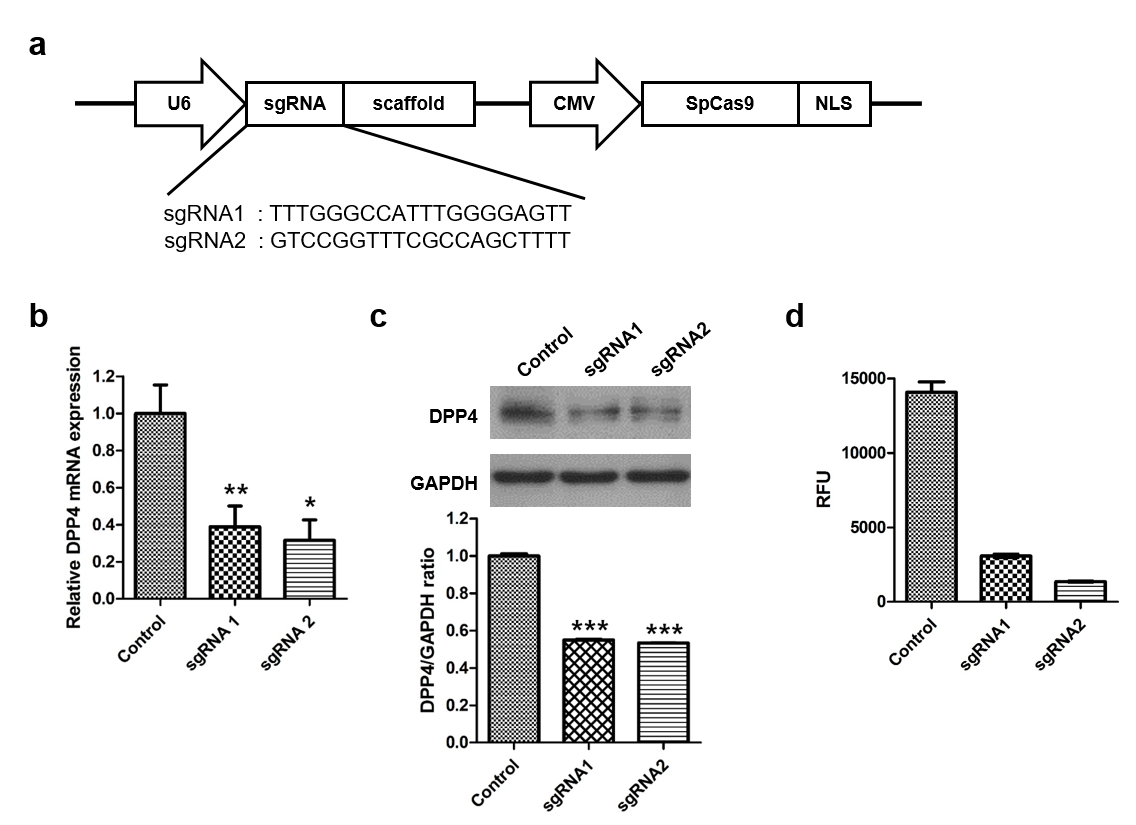
**

**Figure S1.** Design of sgRNA for human DPP4 gene targeting. (**a**) Schematic for a Cas9/sgRNA plasmid. We designed two different sequences of sgRNAs for human *DPP-4* gene. (**b**) The expression level of *DPP-4* mRNA was examined using quantitative real-time PCR. Cas9-RNP (sgRNA2) showed lower expression by ~ 69 % compared to that of untreated control cells. (**c**) Corresponding DPP-4 enzyme level was reduced by 46 % based on western blotting after treatment with Cas9-RNP (sgRNA2). (**d**) DPP-4 enzyme activity assessed with an assay kit. n = 3; **P* < 0.05, ***P* < 0.01, ****P* < 0.001.


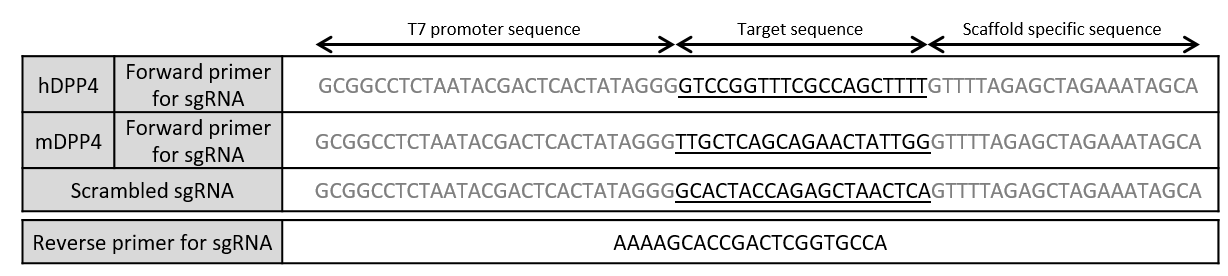


**Table S1. List of primer sequences for *in vitro* sgRNA transcription**. Designed sgRNA sequence for targeting DPP-4 gene region, we prepared sgRNAs from various primer sequences in the list by *in vitro* transcription.


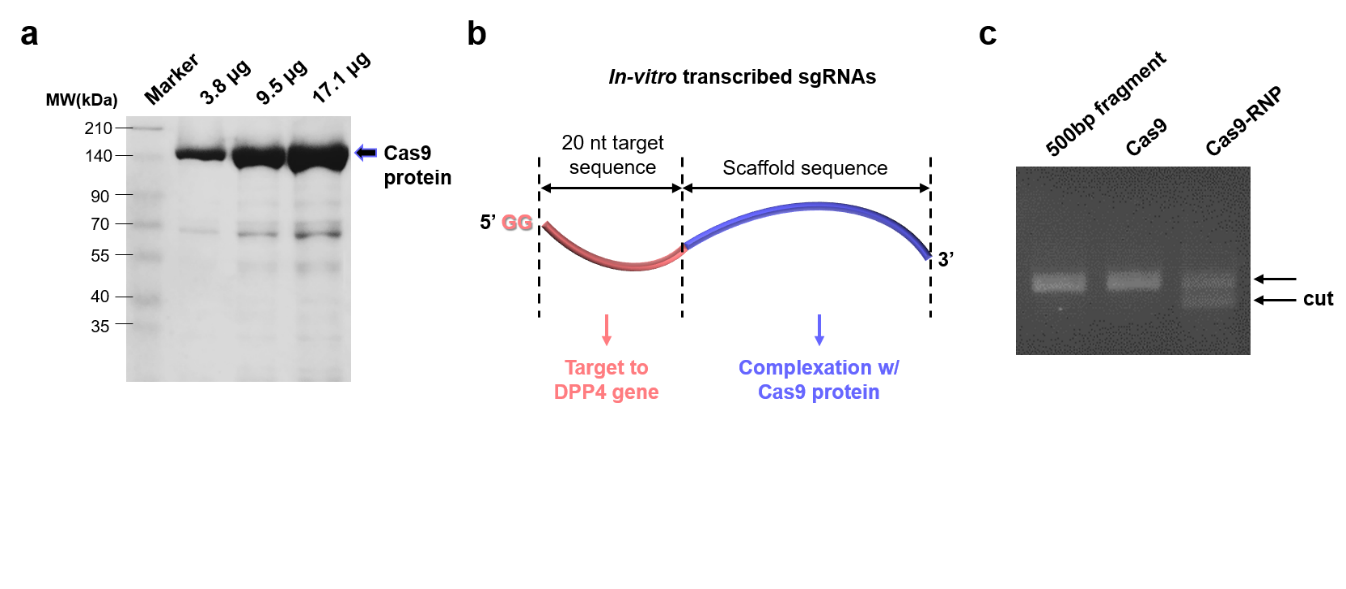


**Figure S2.** Purification and cleavage ability of the prepared Cas9-RNP complex. (**a**) 10 % SDS-PAGE of recombinant Cas9 protein purified from *E.* *coli.* (**b**) Schematic representation of *in vitro*-transcribed sgRNAs composed of a 20-nucleotide guide sequence for *DPP-4* gene recognition and a scaffold sequence for complexing with Cas9 recombinant protein. (**c**) *in vitro* cleavage assay. Cas9-RNP showed clear cleavage of 500-bp target *DPP-4* gene.


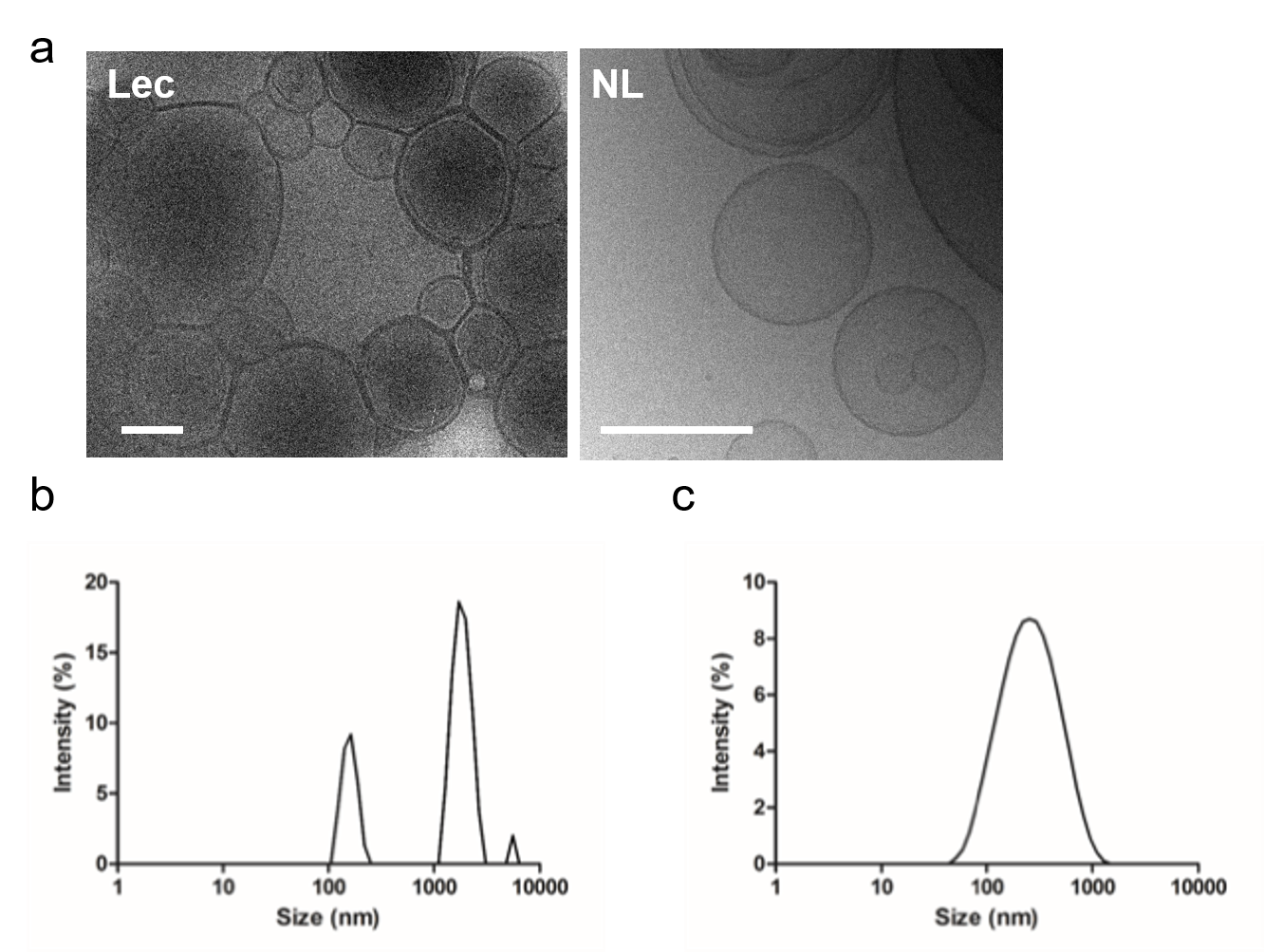


**Figure S3.** Characterization of the nano-liposomal particle. (**a**) Cryo-EM images of lecithin-alone particle (left, Lec) and NL particle (right) containing Cas9-RNP complexes (Scale bar = 200 nm). (**b)** DLS data of Lec@Cas9-RNP. (**c**) DLS data of NL@Cas9-RNP. The diameter of Lec@Cas9-RNP ranged from 164.2 to 1718 nm while NL@Cas9-RNP showed a uniform size distribution with an average diameter of 220.2 nm.

**
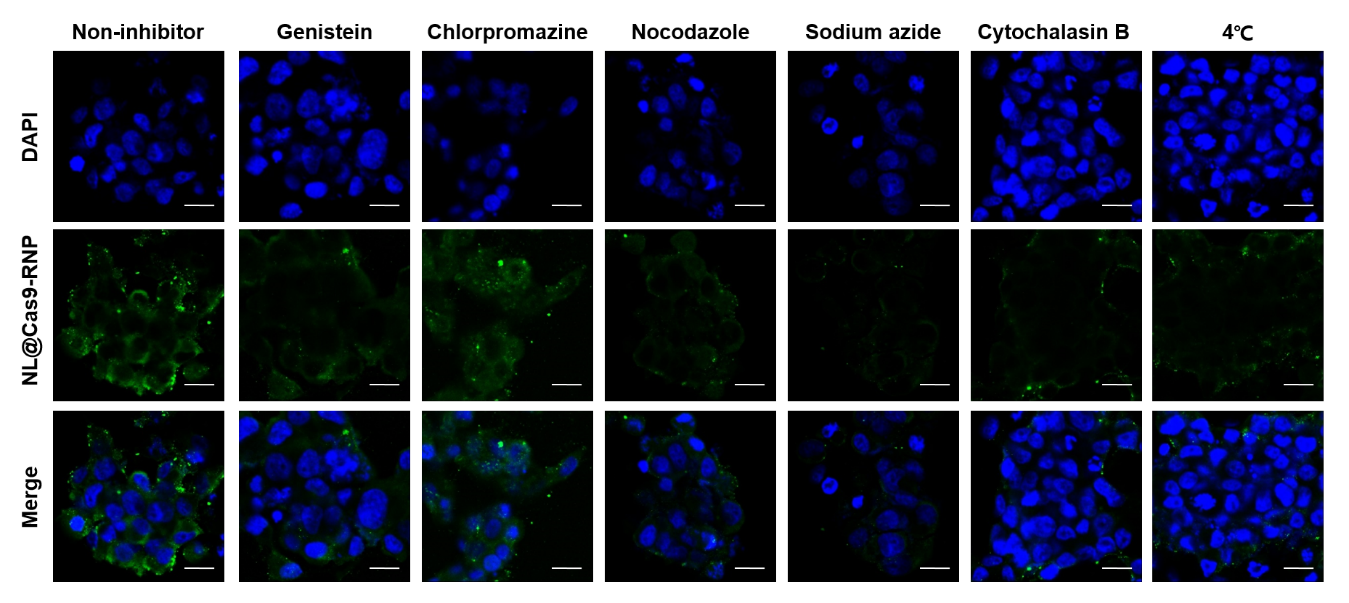
**

**Figure S4.** Uptake mechanism study of NL@Cas9-RNP particle into cells. Assessment of the uptake mechanism using green fluorescence staining of delivered Cas9 protein with anti-Cas9-488 antibodies under various conditions such as inhibitor treatment or culture temperature change. The nucleus is stained with DAPI (blue). SNU398 cells were pretreated for 30 min with various inhibitors: genistein (200 μM), chlorpromazine (30 μM), nocodazole (50 μM), sodium azide (0.01 %), or cytochalasin B (5 μM) at 37 ^o^C or 4 ^o^C before NL@Cas9-RNP treatment (Scale bar = 50 μm).


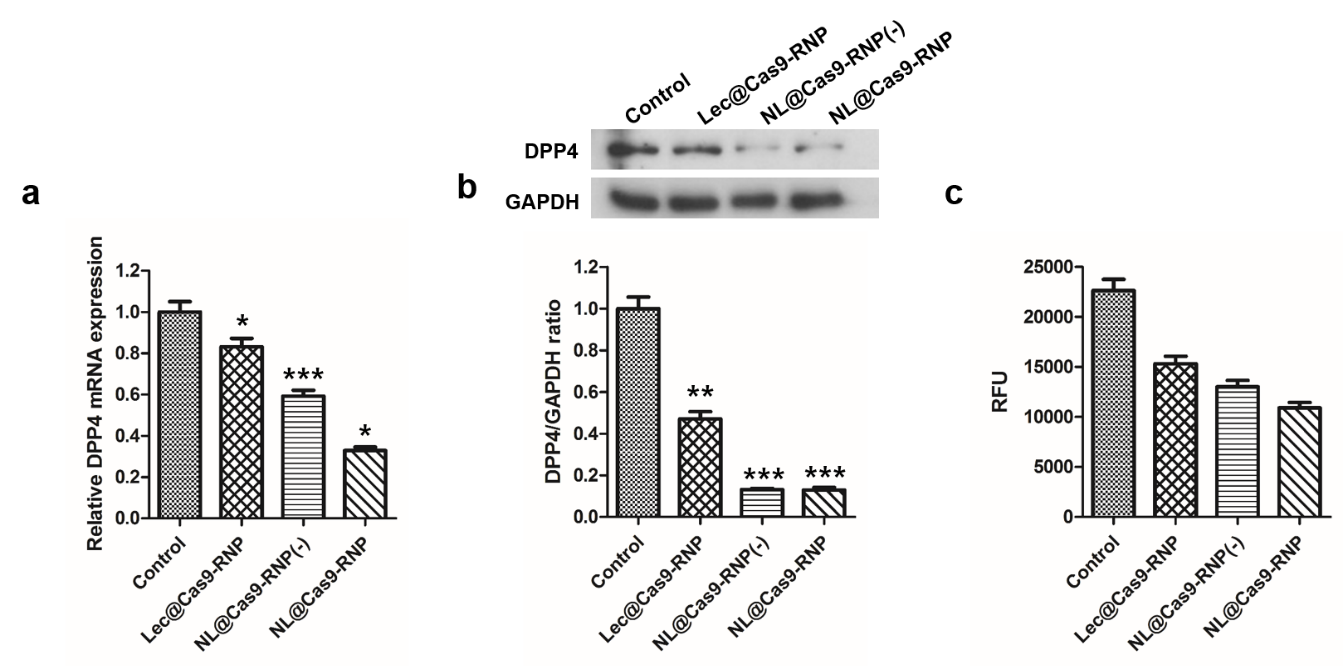


**Figure S5.** Gene editing efficiency in human cells. We investigated whether various nano-liposomes were able to perform gene editing by delivering Cas9-RNP into mammalian cells. SNU398 cells were treated with Lec@Cas9-RNP, NL@Cas9-RNP(-), or NL@Cas9-RNP. Expression levels of *DPP-4* mRNA and protein were measured using quantitative real-time PCR (**a**) and western blotting (**b**), respectively. NL@Cas9-RNP has the highest editing efficiency with decreased mRNA (67 %) and enzyme protein (87 %) expression. In particular, DPP-4 enzyme activity owing to NL@Cas9-RNP delivery was decreased by 48 % compared to that of control SNU398 cells (**c**). n = 3; **P* < 0.05, ***P* < 0.01, ****P* < 0.001.


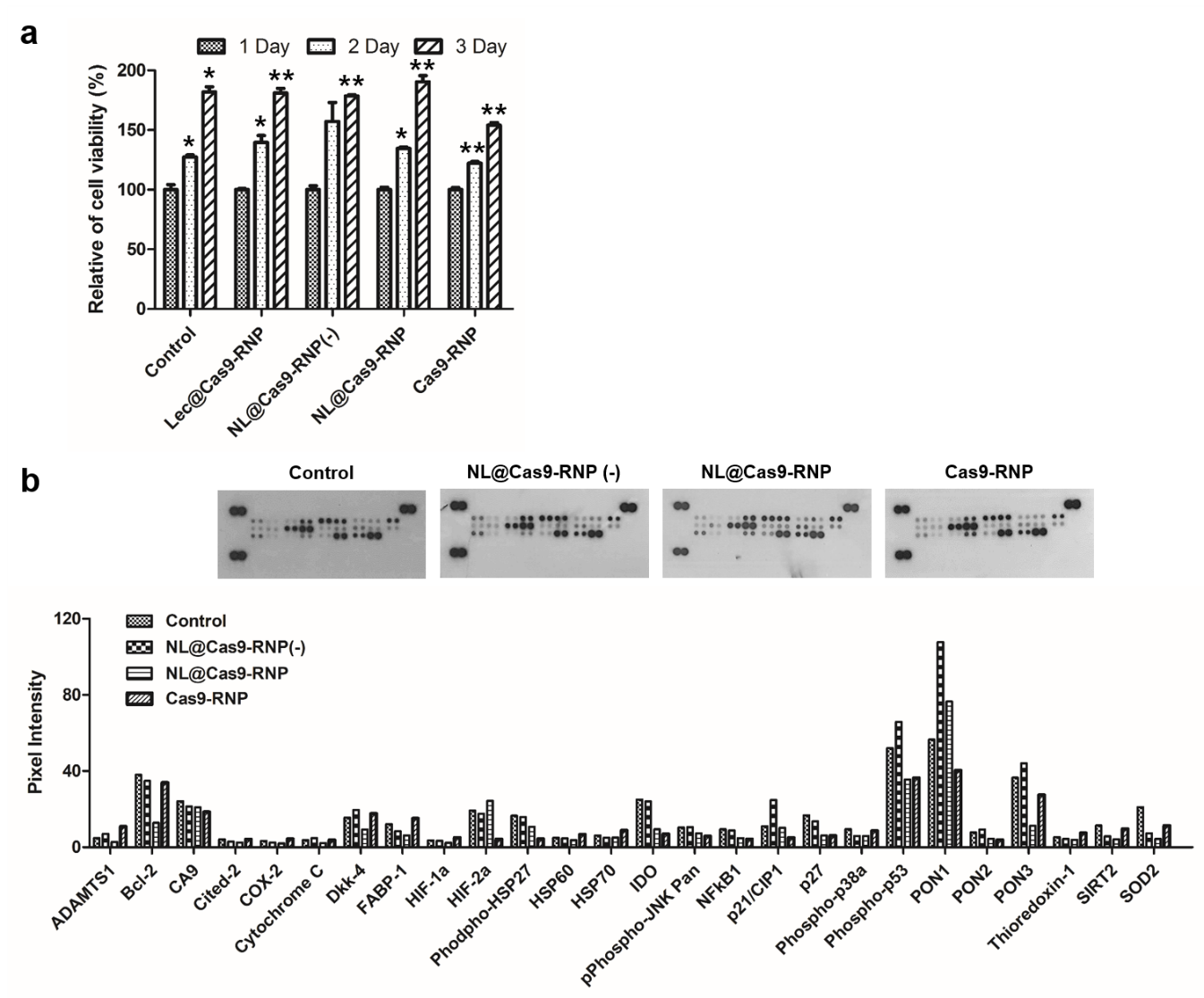


**Figure S6.** Cell toxicity assessment of various particles. (**a**) Cell viability after treatment with various particles containing Cas9-RNP complexes using WST-1 assay. (**b**) Cell stress assessment after treatment using Human Cell Stress Array kit (R&D Systems).


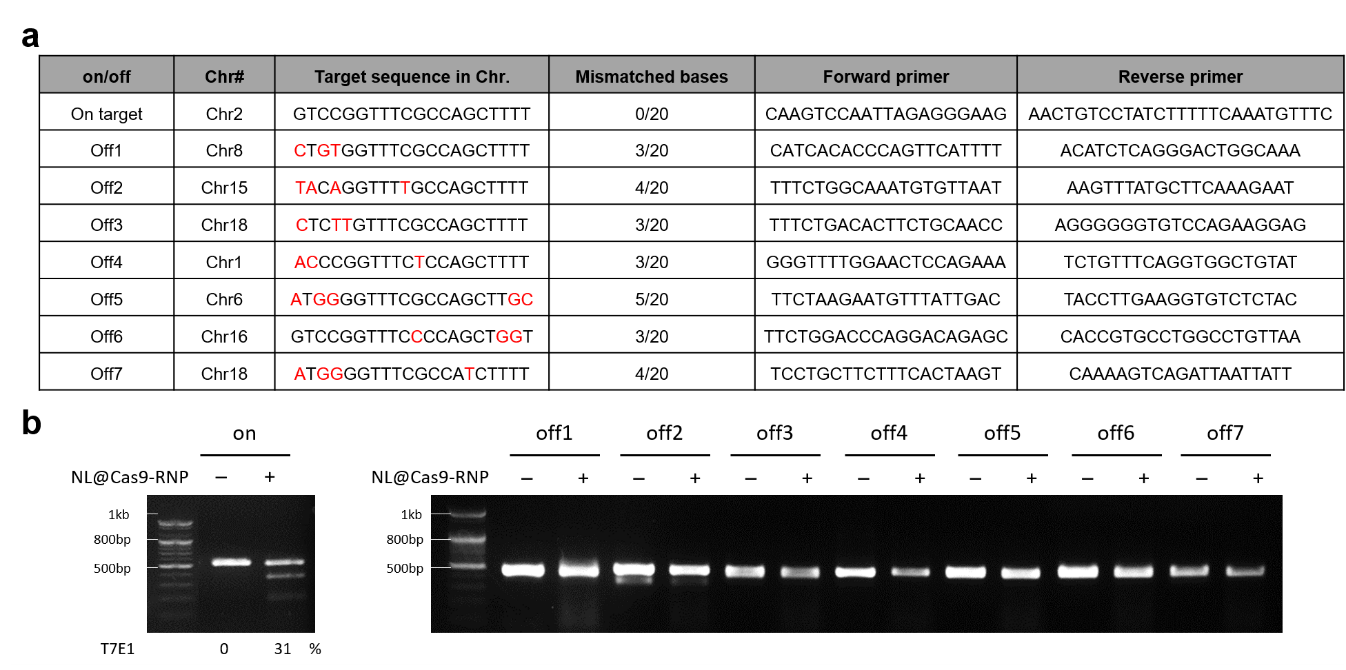


**Figure S7.** Off-target effects of NL@Cas9-RNP. (**a**) List of on- or off-target sequences with mismatch sites and mismatched bases shown in red. (**b**) On-target or potential off-target effects in various target sequence of chromosomes (Chr#) were identified by T7EI assay. Cas9-RNP shows deletion of the DNA on-target site in a cell.


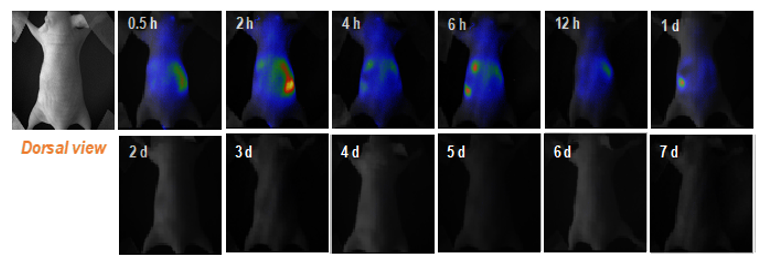


**Figure S8.** *In vivo* monitoring. NIR dye intensity of bare conjugated Cas9 proteins was monitored by optical imaging after injection.

***
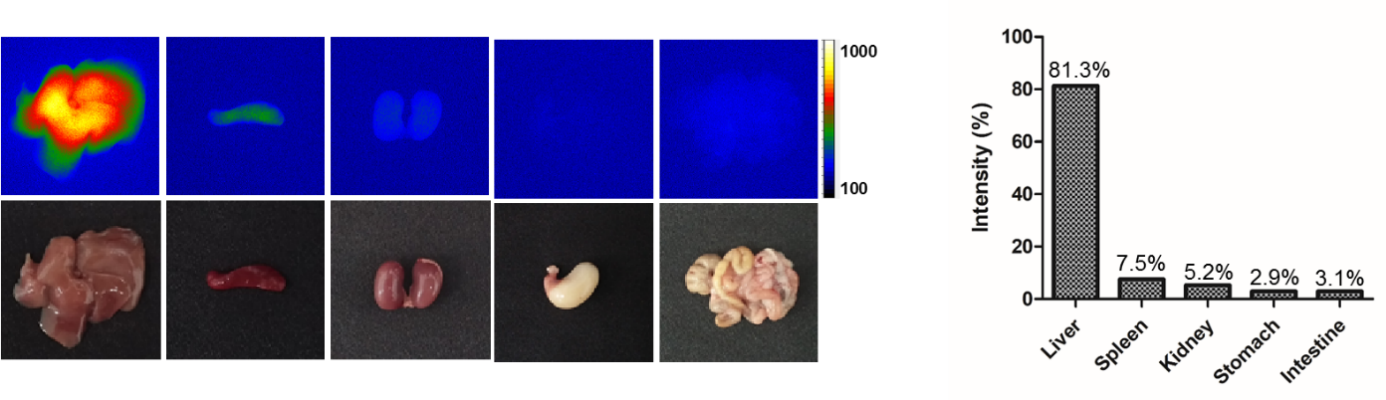
***

**Figure S9.** *In vivo* bio-distribution data. NL particles with dye-labeled Cas9-RNP complexes were used to determine bio-distribution in various organs.


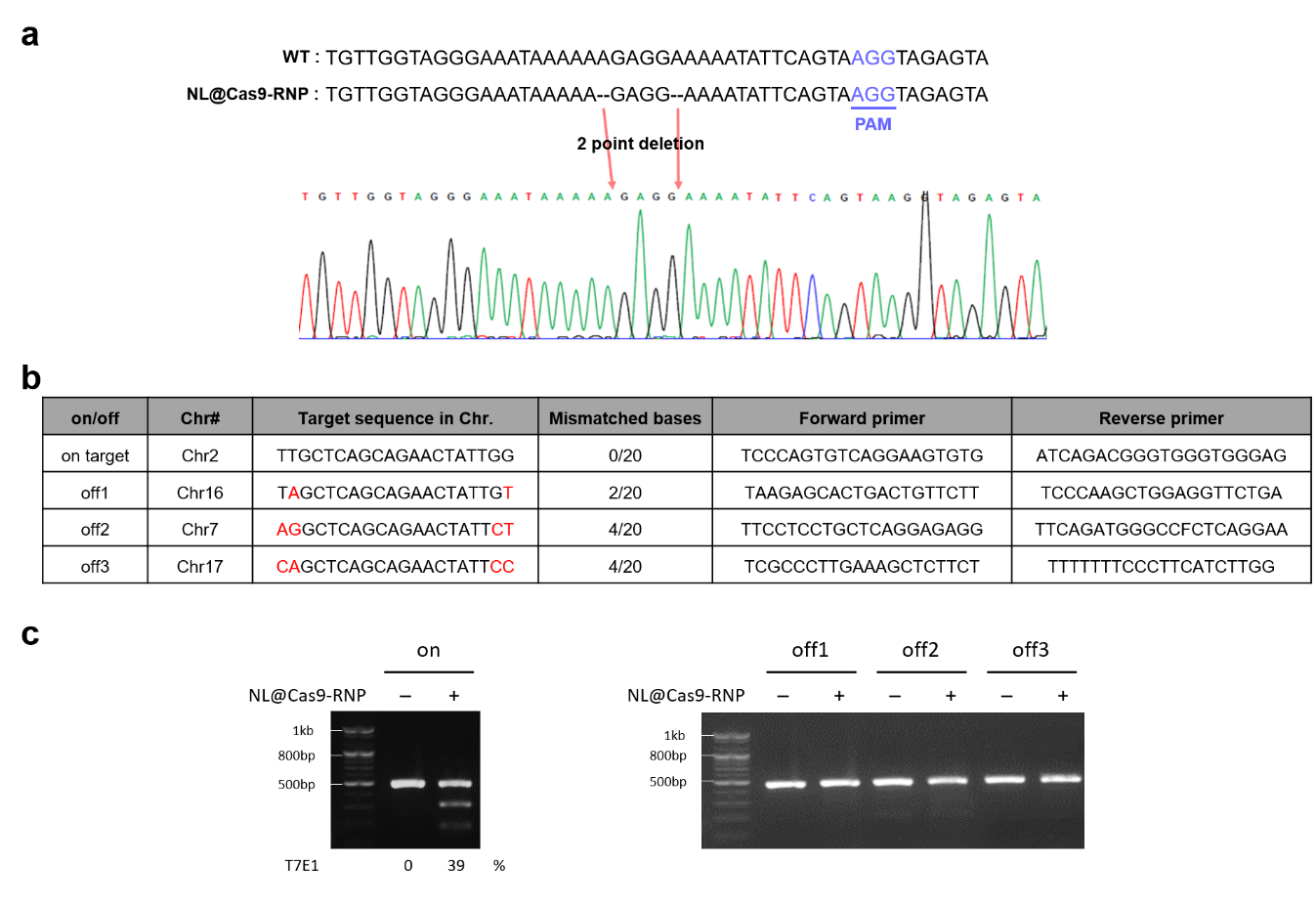


**Figure S10.** *In vivo* gene editing efficiency. (**a**) Representative sequence analysis of deletions at *DPP-4* locus in *db/db* mice after gene editing by Bigdye-terminator sequencing (see Methods). (**b**) List of various sequences of target sequences with mismatch sites and mismatched bases shown in red. (**c**) On-target and potential off-target effects in various target sequence of chromosomes were identified by T7EI assay. NL@Cas9-RNP achieved *in vivo* gene editing efficiency of 39 % for DNA on-target site with low off-target effect in mouse liver.

**
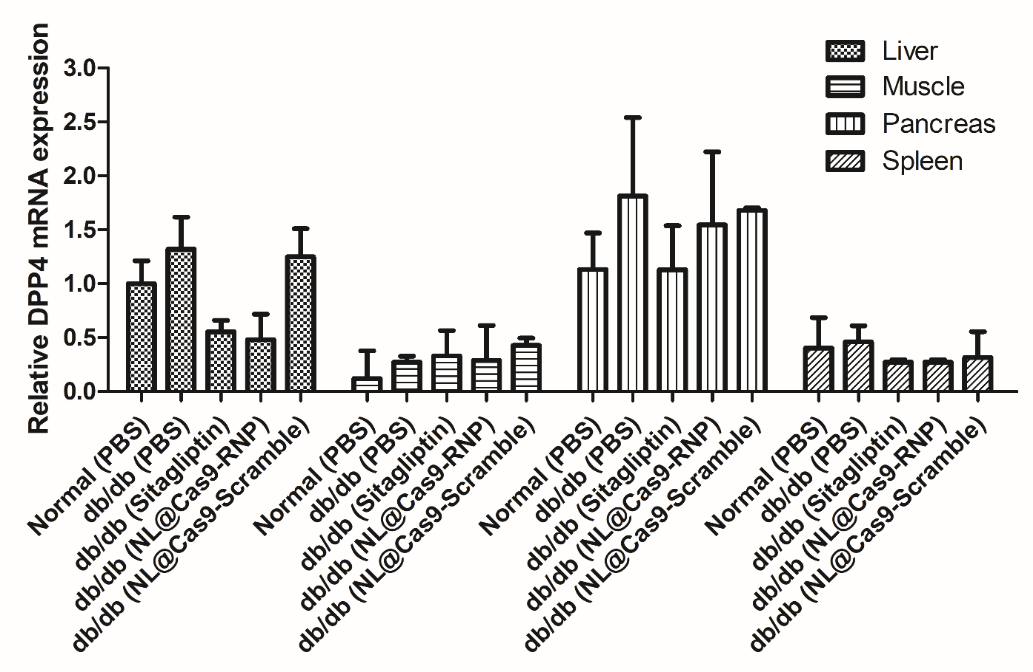
**

**Figure S11.** Comparison of *DPP-4* mRNA expression distribution in various organ tissues of *db/db* mice after treatment based on quantitative real-time PCR.
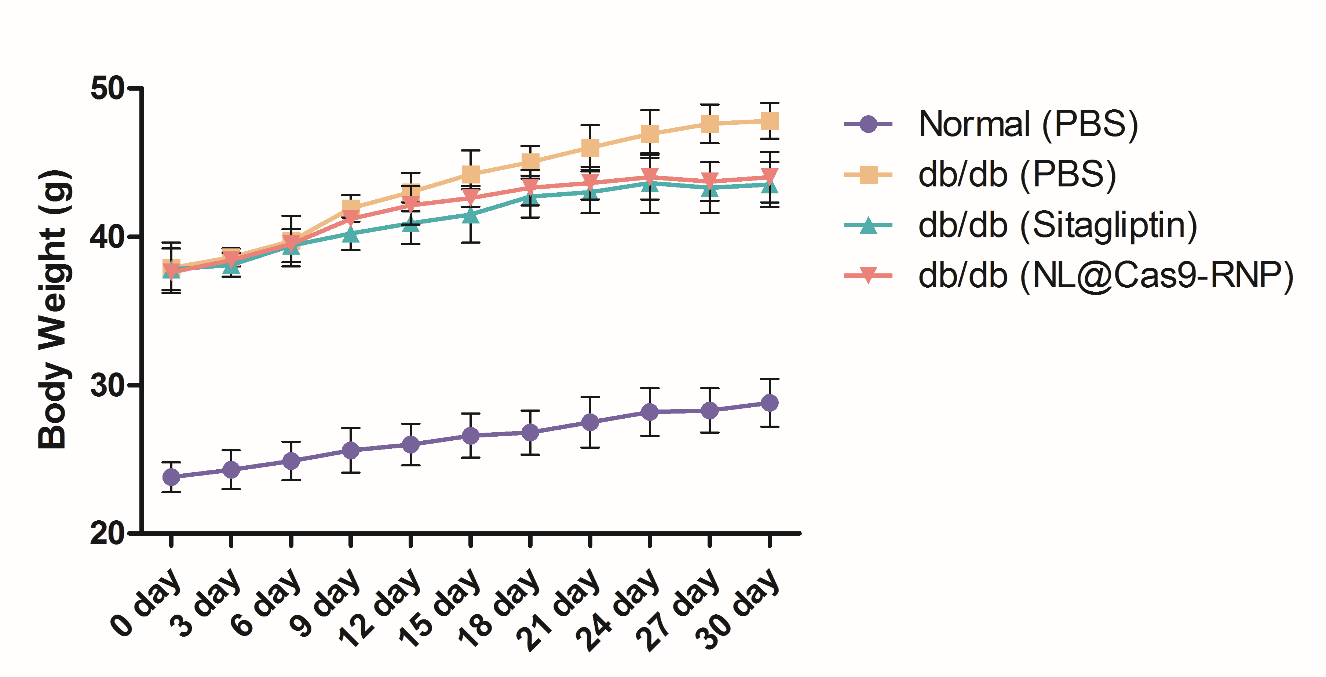


**Figure S12.** Sitagliptin and NL@Cas9-RNP treated mice had relatively decreased body weight compared to control mice.
